# Photodynamic and Photothermal Therapies using Nanotechnology Approach in Alzheimer's Disease

**DOI:** 10.2174/011570159X370790250317045223

**Published:** 2025-04-15

**Authors:** Büşra Öztürk, Huriye Demir, Mine Silindir Günay, Yagmur Akdag, Selma Sahin, Tugba Gulsun

**Affiliations:** 1 Department of Pharmaceutical Technology, Faculty of Pharmacy, Hacettepe University, Ankara, Türkiye;; 2 Department of Pharmaceutical Technology, Faculty of Pharmacy, Ondokuz Mayıs University, Samsun, Türkiye;; 3 Department of Radiopharmacy, Hacettepe University, Ankara, Türkiye

**Keywords:** Alzheimer’s disease, drug delivery systems, nanotechnology, nano theranostics, photodynamic therapy, photothermal therapy

## Abstract

Alzheimer's disease is a neurodegenerative disease that impairs cognitive function. The incidence of Alzheimer's disease increases with the increase in the elderly population. Although the clear pathogenesis of Alzheimer's disease is not yet known, the formation of amyloid plaques and tau fibrils, diminished acetylcholine levels, and increased inflammation can be observed in patients. Alzheimer's disease, whose pathogenesis is not fully demonstrated, cannot be treated radically. Since it has been observed that only pharmacological treatment alone isn’t sufficient, alternative approaches have become essential. Among these approaches, nanocarriers greatly facilitate the transport of drugs since the blood-brain barrier is an important obstacle to the penetration of drugs into the brain. Photosensitizers trigger activation after exposure to near-infrared radiation light of a suitable wavelength or laser light, resulting in the selective destruction of Aβ plaques. Photodynamic therapy and photothermal therapy have been investigated for their potential to inhibit Aβ plaques through photosensitizers. By ThT fluorescence measurements, TAS-loaded Ce6 micelles show inhibiting Aβ monomers from formation Aβ aggregates and degradation of protofibrills to small fragments. By using these photosensitizers, near-infrared radiation fluorescence imaging can be used as a theranostic. In this review, potential treatment options for photodynamic therapy and photothermal therapy for Alzheimer's disease are summarised, and a simultaneous or combined approach is discussed, taking into account potential nanotheranostics.

## 1 INTRODUCTION

Alzheimer's Disease (AD) is a progressive neurodegenerative disorder whose prevalence is increasing worldwide in older people with impairments in short-term memory, verbal fluency, visuospatial processing, and executive functions (mental agility) [1]. After the diagnosis of AD, the average survival time of patients varies from 2.3 to 8 years [2]. AD is the most common cause of dementia and the fifth leading cause of death in people over the age of 65. The total healthcare cost of treating AD and related dementias is projected to rise $16.9 trillion in 2050 as the proportion of elderly people in the population increases [3].

According to dementia prevalence estimates for 204 countries, there were 57.4 million people with dementia globally in 2019 [4]. This number is projected to rise to 152.83 million by 2050. Additionally, a study on age-standardized prevalence rates for AD and other dementias has found that Türkiye has the highest age-standardized prevalence [5].

The exact treatment for AD has not yet been discovered. Consequently, numerous hypotheses have been proposed to explain the pathophysiology of AD. Based on these hypotheses, many drugs have been tested as monotherapies, in combination, or with additional procedures to overcome the challenges involved. However, many small molecules and large molecules are unable to cross the blood-brain barrier (BBB). To address this issue, nanocarriers have been developed to facilitate crossing the BBB. Additionally, some auxiliary techniques, such as ultrasound, radiation, and light, are being explored along with drug delivery systems. Among these approaches, theranostic studies attract attention for their potential to enable both diagnosis and treatment.

## 2 HYPOTHESES FOR THE PATHOPHYSIOLOGY OF ALZHEIMER'S DISEASE

Although the pathophysiology of AD has not been fully understood, studies have shown that it is a series of events that cause or accelerate the formation of each other. It can be described as a sequence of events that begins with gene mutations, unusual amyloid plaque development, oxidation, abnormal neurofibrillary tangles (NFT) formation, causes significant disturbances in the release or uptake of specific neurotransmitters, excitotoxicity, and ends with neuronal death. Several explanations have been proposed to explain AD. The most common theories are as follows:

- Aβ and hyperphosphorylated tau protein hypothesis.
- Oxidative stress hypothesis.
- Cholinergic hypothesis.
- Excitotoxic hypothesis.

### 2.1 Aβ and Hyperphosphorylated Tau Protein Hypothesis

#### 2.1.1 Aβ Hypothesis

Aβ 40&42 amino acid peptides are generated from Aβ precursor protein (APP), which is closely associated with memory and neuronal plasticity [6]. The APP protein is broken down and metabolized by proteolytic enzymes. These enzymes are known as α, β, and γ-secretases. When APP is degraded *via* α-secretase enzymes, αAPP and C83 are produced, both of which are neuroprotective compounds [7]. When APP is degraded by β-secretase enzymes and then γ-secretase carries out the proteolysis of APP, this reaction results in increased levels of C83 and C99AP in the central nervous system (CNS) and produces the β-sAPP fragment. The β-sAPP fragment is cleaved by γ-secretase and generates the Aβ-42, which accumulates and provides for microglial activation and neuroinflammation [7, 8].

Numerous factors influence the clearance of Aβ peptides. Of particular importance is the genetic predisposition of people with Alzheimer's disease. These predisposing factors include presenilin polymorphisms (presenilin1 and presenilin2), apolipoprotein E4, PICALM, and APOJ genes [9-11].

One of the leading pathological changes in AD is the development of amyloid plaques, which can result from increased Aβ peptide production or an impairment in its clearance [12].

#### 2.1.2 Tau Protein Hypothesis

Tau protein is a microtubule-associated protein essential for maintaining neuronal stability in the CNS. Evidence suggests that tau protein aggregates are present in the brain tissue of individuals with AD [13, 14]. It has also been reported that kinase overload triggers the hyperphosphorylation of this protein in patients with the cognitive decline characteristic of AD. As a result, the tau protein dissociates from microtubules and initiates a process that damages cells and ultimately leads to cell death [15]. Neurofibrillary tangles, mediators of neurodegeneration and dysfunction in AD patients, are formed when phosphorylated tau protein aggregates [16].

### 2.2 Oxidative Stress Hypothesis

The brain consumes 20% more oxygen than other body tissues. This makes it more susceptible to oxidative stress [17], which has been mentioned in the pathogenesis of AD [18]. Evidence also suggests that the brains of AD patients have higher levels of iron, aluminum, and mercury, which are metals known to create free radicals. The development of amyloid plaques and NFT is thought to be facilitated by oxidative stress, 4-hydroxynonenal, oxidative damage, lipid peroxidation of proteins and DNA, malondialdehyde, advanced glycation end products (AGEs), peroxynitrite, carbonyls, hemoxygenase-1, and SOD-1 levels [19].

### 2.3 Cholinergic Hypothesis

The cholinergic hypothesis relates to the loss of acetylcholine (Ach) neurons and enzymatic function in the CNS. It is the oldest theory put forward to interpret the etiology and mechanism behind AD-related neuropsychiatric symptoms [20]. The progression of AD is also associated with decreased M2 muscarinic acetylcholine receptors (mAChRs) and a7 or a4b2 nicotinic acetylcholine receptors (nAChRs) in pre- and postsynaptic neurons [21, 22].

### 2.4 Excitotoxic Hypothesis

The excitatory neurotransmitter glutamate is engaged with synaptic pliancy, processes connected to memory and learning, and other functions in the brain [23]. Due to their involvement in memory formation and modulation processes, glutamate N-methyl-D-aspartate (NMDA) receptors are critical in the progression of cognitive disorders, particularly AD [24, 25].

Inadequate recycling of glutamate released in the synaptic gap leads to hyperexcitability of the channels of these receptors in AD. Through these mechanisms, the neurotransmitter continues to act moderately and chronically on the receptors, triggering them and causing an excessive influx of calcium ions (Ca^2+^) into the neuron. A few organelles, including the mitochondria and the endoplasmic reticulum, are very sensitive to variations in the intracellular calcium ion concentration. The increased intracellular calcium ion concentration harms the cells and results in cell deterioration and death [24, 26].

## 3 APPROVED DRUGS FOR ALZHEIMER'S DISEASE BY LEGAL AUTHORITIES

Current therapies for AD aim to delay clinical degeneration and enhance cognition and function, even though there is no known cure. Additional therapies may help relieve symptoms such as disorientation and memory loss. In the US, drugs that may slow the clinical decline of AD patients, as well as treatments that may temporarily relieve some of the symptoms associated with the disease, have been approved by the Food and Drug Administration (FDA) (Table **1**) [27].

Current pharmacotherapy for AD falls into distinct categories: acetylcholinesterase inhibitors rivastigmine, donepezil, galantamine, and an NMDA antagonist, memantine. Rivastigmine, marketed under the brand name Exelon^®^, is a cholinesterase inhibitor approved by the FDA in 2000 for the treatment of AD and Parkinson's disease [28]. Its pharmacokinetics and pharmacodynamics demonstrate a dose-response correlation, with increased efficacy observed at higher doses [29]. Rivastigmine can be administered orally or *via* a transdermal patch. Clinical trials have confirmed its benefits in various stages of AD.

Donepezil, marketed under the brand name Aricept^®^, is a pioneering second-generation non-competitive, reversible acetylcholinesterase (AChE) inhibitor. It received FDA approval in 1996, expanding its use in the treatment of mild, moderate, and severe AD dementia. As one of the most frequently prescribed drugs for AD patients, its widespread use highlights its clinical significance [30].

Galantamine, marketed as Razadyne^®^, was approved by the FDA in 2001 for the treatment of mild to moderate AD. It is characterized by good tolerability and is recognized for its ability to improve functionality, cognition, and daily activities in patients with mild-to-moderate AD in a relatively short period of time, usually around 6 months. Additionally, the use of this drug has been associated with delayed Behavioral and Psychological Symptoms of Dementia (BPSDs), leading to reduced caregiver burden [31].

Memantine, marketed as Namenda^®^, obtained FDA approval in 2003 for the treatment of moderate-to-severe AD. Excitotoxicity results from excessive stimulation of glutamate receptors, leading to neuronal damage due to elevated intracellular calcium levels. Memantine, a non-competitive NMDA receptor antagonist, attenuates the adverse effects of elevated brain glutamate levels [32]. When administered orally, memantine is efficiently absorbed, and its pharmacokinetics remain linear at typical therapeutic doses [33].

Apart from these approved drugs, the FDA granted accelerated approval for aducanumab on June 7, 2021 [34]. Aducanumab (Aduhelm^TM^) is an anti-amyloid antibody and is administered by intravenous infusion. It is a high-affinity, fully human immunoglobulin gamma 1 (lgG1) monoclonal antibody (mAb) that binds to aggregated forms of Aβ and predominantly binds to parenchymal amyloid compared to vascular amyloid. It has been reported that intraperitoneal injection of aducanumab into Tg2576 mice detected parenchymal plaques and facilitated their clearance without causing microbleeds [35]. It crosses the BBB and selectively binds to the soluble oligomers and insoluble fibrillar conformations of Aβ-plaques within the brain, thereby preventing disease advancement. This specific binding sets it apart from other immunotherapeutic agents targeting Aβ [36, 37]. The manufacturer of aducanumab announced plans to discontinue the development and commercialization of aducanumab in 2024 for reasons unrelated to safety or efficacy [38].

The accelerated approval granted by the FDA on January 6, 2023, for lecanemab, marketed under the brand name Leqembi, was converted to traditional approval on July 6, 2023 [39]. The removal of amyloid and its promising potential for clinical benefits are supported by evidence from the phase 2 trial [40, 41]. Lecanemab is a humanized IgG1 antibody originating from mAb158 that exhibits selective binding to soluble Aβ protofibrils [42]. This drug reduces Aβ-fibril aggregation within astrocytes, consequently reducing amyloid plaques, resulting in clinical benefits and disease modification. It has been tested in people with early AD and has demonstrated efficacy in decreasing markers of amyloid deposition. Over 18 months, the decline in measures of cognition and function was moderately less than placebo [43]. Leqembi has been refused marketing authorization in the EU by EMA. The reason for this decision is that the drug’s effect on delaying cognitive decline is small and does not outweigh the risks associated with serious side effects, particularly amyloid-related imaging abnormalities (ARIA). This side effect can lead to serious health issues in some patients, and the risk is more pronounced in individuals predisposed to Alzheimer’s disease. Therefore, the Committee for Medicinal Products for Human Use concluded that the benefits of the treatment do not outweigh the risks [44-47].

One of the main essential difficulties encountered in the therapy of neurodegenerative disorders that occur in the brain, such as AD, is the inadequate delivery of drugs to the brain and the need to reach a specific region and ensure long-term release. This is related to very tight barriers, including the blood CSF barrier and the BBB.

## 4 BRAIN BARRIERS

CNS homeostasis is crucial for the proper functioning of brain cells. The brain barriers contribute to CNS homeostasis by protecting the brain from fluctuations in the blood component concentrations and facilitating the transport of nutrients into the brain and metabolic waste products out of the brain. Two main barriers separate the CNS from the periphery: the BBB and the CSF barrier [48].

### 4.1 Blood Brain Barrier (BBB)

The BBB allows Aβ to pass from the brain into the peripheral circulation. Specifically, reduced levels of LRP-1 (low-density lipoprotein receptor-related protein 1) and increased levels of RAGE (receptor for advanced glycation end products) at the BBB can disrupt Aβ transport [49, 50]. The AD pathogenesis is associated with various structural components of the BBB, including pericytes, astrocytes, vascular endothelial cells, and tight junctions. BBB dysfunction triggers neuroinflammation and oxidative stress, leading to increased activity of β-secretase and γ-secretase, ultimately promoting Aβ production [49, 50]. The progressive accumulation of Aβ in the brain and BBB dysfunction may create a feedback loop contributing to cognitive decline and the onset of dementia [51]. The correlation between BBB dysfunction and tau pathology has been extensively documented. Thus, modulating BBB function could offer a novel therapeutic approach to AD treatment [52].

The properties required for the drug transport across the BBB include low albumin binding of the drug, less than eight hydrogen bonds, molecular weight less than 500 Da, neutral pH, between 7.5-10.5 pKa values, and close to 2.1 logP value [53, 54]. Methods of drug transport to BBB can be separated into invasive and noninvasive methods. Invasive methods are based on surgical applications, direct injection into cerebral arteries, and reversible disruption of the BBB. However, this application is risky as it may enhance the delivery of unwanted toxic substances to the CNS in addition to therapeutic or diagnostic molecules. In noninvasive methods, the therapeutic molecule or imaging agent is targeted to the brain *via* a nanocarrier system containing the main molecule and a penetrating or targeting molecule, or it can be delivered to the brain *via* a biological route such as intranasal administration. In addition to being more reliable, non-invasive methods are generally preferred [55, 56]. Nearly all biological drugs and over 98% of small drug molecules cannot pass the BBB [57]. Thus, developing delivery mechanisms capable of penetrating this barrier is critical for treating and imaging neurodegenerative disorders such as AD [58]. Generally, there are five distinct transport mechanisms for molecules across the BBB: (I) receptor-mediated transcytosis, (II) adsorptive-mediated transcytosis, (III) diffusion, (IV) carrier-mediated transcytosis, and (V) paracellular diffusion [58].

### 4.2 Blood Cerebrospinal Fluid Barrier

CSF is produced by ependymal cells in the ventricles of the brain. The main function of the CSF is to provide mechanical and immunological protection [59].

The blood-CSF barrier is essential in regulating the brain environment, similar to the BBB. However, its surface area is much smaller than that of the BBB [60]. Ions and small molecules, including vitamins and nutrients, can cross the CSF relatively easily. In contrast, larger substances such as cells, proteins, and glucose are restricted from crossing [61].

The breakdown of the blood-CSF barrier is a relatively newer concept compared to the well-established BBB breakdown, which has been extensively studied in various neurological disorders, including AD. CSF is produced by the choroid plexus (CP), which comprises ciliated epithelial cells, connective tissue, and fenestrated blood vessels located in the brain's ventricles. While these fenestrated vessels allow for high permeability, tight junctions on the surface of the epithelial cells prevent harmful blood-borne substances from entering the ventricular system, thus forming the Blood CSF Barrier. Sodium, chloride, and bicarbonate ions actively flow into the ventricular system through ion channels and cotransporters at the Blood CSF Barrier, creating an osmotic gradient that pulls in water. Aging leads to the formation of inclusions called Biondi bodies within CP epithelial cells and the thickening of basement membranes, which may reduce CSF secretion by the CP [62].

Substances that cannot cross the blood CSF barrier required by the brain can be transported into the CSF by the CP cells using ATP [63]. People with Alzheimer's disease have been found to have reduced amyloid (Aβ_1-42_) and increased phosphorylated tau (*p*-tau_181p_) and total tau (*t*-tau) in the CSF [64].

## 5 NANOTECHNOLOGICAL APPROACHES IN ALZHEIMER'S DISEASE TREATMENT

**Fig. (1):**
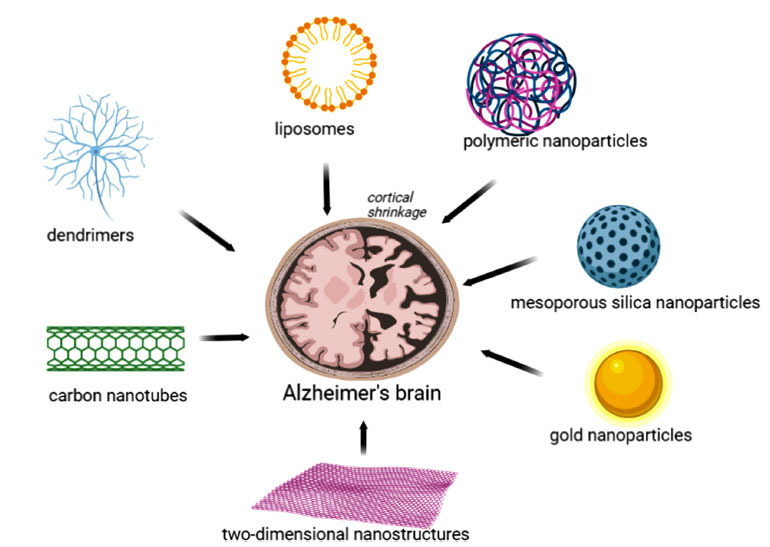
Nanotechnology-based drug delivery systems approach for the diagnosis, imaging, and treatment of Alzheimer's disease. Created using Biorender.

The nano-based therapeutic systems (nanocarriers) typically range from 1 to 1000 nm and comprise materials such as inorganic materials, lipids, polymers, and different structures (Fig. **1**). Nanocarriers entered the field of research in the late 1960s when Peter Paul Speiser developed the first nanoparticles [65]. In the 1970s, Georges Jean Franz Köhler and César Milstein succeeded in producing monoclonal antibodies [65]. In the present, various types of nanocarriers have been developed, such as liposomes, micelles, dendrimers, carbon nanotubes, *etc*.

Nanocarriers have advantages such as enhanced drug efficacy, targeted delivery of diagnostic agents, targeting, reduced side effects, increased patient compliance, controlled release, enhanced stability, optimized drug loading, pharmacokinetic/pharmacodynamic improvement, modifiability, hydrophilic/hydrophobic drug encapsulation, and enhanced bioavailability [66].

To enable imaging, diagnosis, and therapy of AD, research has focused on developing efficient surface design methods for delivering nano-based systems across the BBB. Aβ and tau accumulation serve as suitable biomarkers. Disease progression can be evaluated by nuclear imaging of these biomarkers. The use of novel imaging modalities, such as optical imaging, and the development of novel and specific diagnostic agents can contribute to the understanding of AD progression, which may facilitate early therapy [67-69]. The therapy of AD is aimed to reduce Aβ production and agglomeration, along with tau phosphorylation and aggregation into NFTs (Table **2**) [70, 71].

## 6 PHOTODYNAMIC AND PHOTOTHERMAL THERAPY AND MECHANISMS

Photodynamic therapy (PDT) and photothermal therapy (PTT) are both light-activated treatments that target biomaterials or cells. PDT uses photosensitizers (PSs) to produce reactive oxygen species (ROS), free radicals, or singlet oxygen (^1^O_2_), when exposed to light. This process effectively damages target cells or biomaterials.

PTT uses PSs to generate localized heat upon light activation. This increase in temperature can lead to damage to biomaterials and cell destruction and is particularly effective in many cancer treatments. Photosensitizers are important in both therapies because they absorb light at specific wavelengths and convert this energy into a form that will have a therapeutic effect. PDT was first studied as a bactericide with its accidental discovery by Oscar Raab in the 1900s [81]. The modern era of PSs began with their entry into medical use in the 1960s when Lipson and Baldes [82] showed that neoplastic tissue containing a mixture of porphyrins was fluorescent under UV light [83]. In the 1990s, the first FDA-approved photofrin set many standards for clinical practice around the world and formed the basis of modern PDT [84, 85]. Indocyanine green (ICG) is the only chromophore approved by the FDA for clinical imaging and diagnosis, liver function tests, surgical navigation, and ophthalmic angiography [86, 87].

Since then, a lot of research has been done on the use of PDT and PTT, mainly for the treatment of tumors but also for infections, dermatology, and neurodegenerative diseases (Table **3**) [86, 88-93]. PTT can be used for the therapy of different types of cancer and infectious diseases by using near-infrared radiation (NIR) light for maximum tissue penetration and converting energy into heat to kill cancer cells or bacterial infections [94, 95]. Additionally, PTT can also be used for inducing local heat enhancement and promoting drug release from drug delivery systems at the disease site [96-98].

**Fig. (2):**
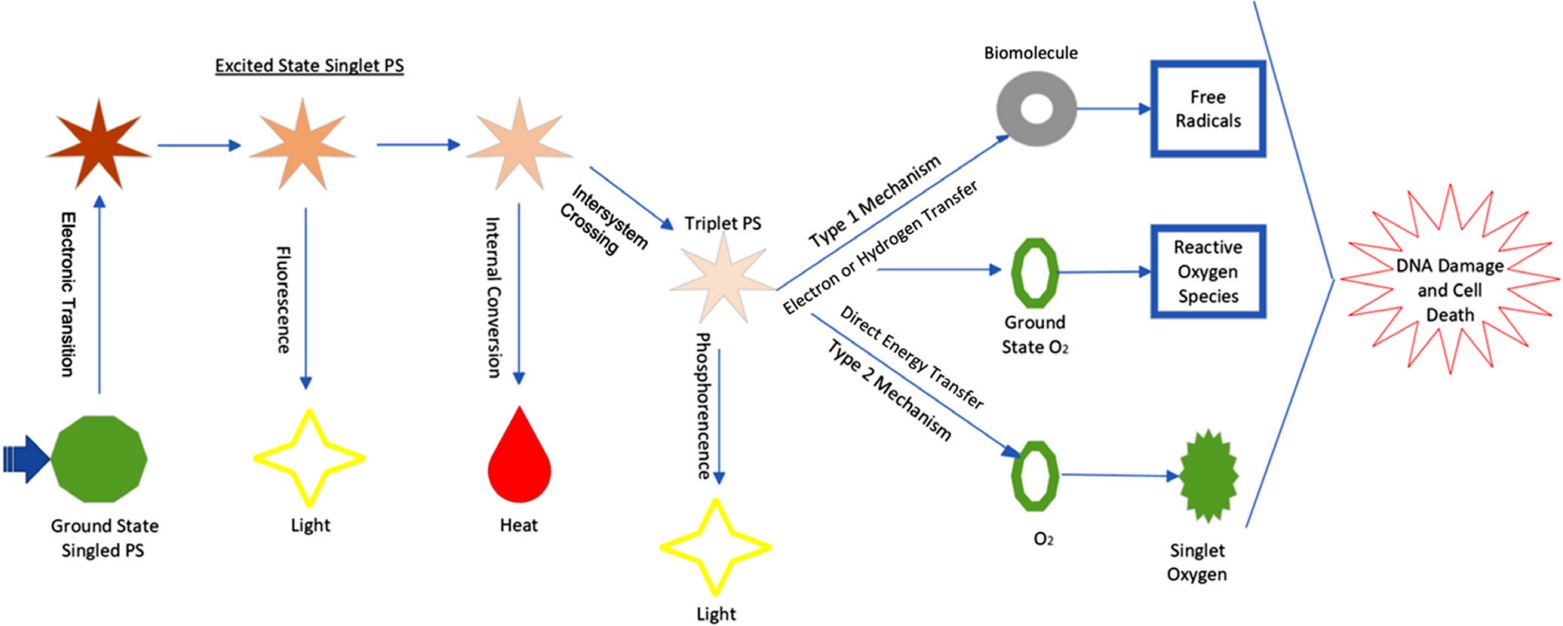
Mechanism of the photodynamic reaction. Reprinted [101-103].

The mechanism of PDT can be explained by the fact that when PS is excited by a light having a suitable quantum energy absorption, it changes from the ground singlet state to an excited singlet state due to photon absorption. This structure is very unstable. It emits heat or fluorescence. It passes through the inter-crossing process and converts to the excited triplet state [99]. In the Type I mechanism, the energy of the excited triplet state of PS can be transferred to a biomolecule containing hydrogen or an electron, forming a free radical. The electrons can interact with oxygen in the basic energy state to form ROS [100]. In the Type II mechanism, the excited triplet state of PS transfers its energy directly to O_2_. This produces singlet oxygen with very strong oxidizing properties (Fig. **2**) [101].

The mechanism of PTT can be explained by providing light-induced localized hyperthermia. Photon energy can be absorbed by PSs and transformed into heat. PTT may be a promising treatment option for AD due to its advantages, such as being inexpensive, having low adverse effects on healthy tissues, and having high spatiotemporal accuracy. NIR light has a longer wavelength and deeper penetration than UV and visible light. These features make NIR light more effective on deeper-seated amyloid. Considering amyloid aggregate formation is affected by temperature change like any other protein complex, local temperature can limit Aβ aggregation, which has a significant potential to change the course of AD.

PS entered medical use with hematoporphyrin and ICG, which are mentioned above. Today, many types and features of PS, such as phthalocyanines, squaraines, and subtraction/substitution structured porphyrins, are used in studies [88].

Porphyrins have become one of the most extensively studied organic dyes for PDT of tumors due to their excellent singlet oxygen generation quantum yield and favorable biocompatibility.

Existing Aβ PSs encompass various categories, including molecules, carbon-based nanomaterials, two-dimensional nanomaterials, metal nanoparticles, polymeric nanoparticles, upconversion nanoparticles (UCNPs), and photoelectrode materials [104].

PSs can be activated with laser light or NIR, which is a light having a wavelength about between 700 nm and 1000 nm. This makes it easier to achieve the necessary penetration into the brain.

### 6.1 Photodynamic Therapy Studies in Alzheimer's Disease

It was observed that the use of UV and visible light in the PDT generally caused shallow penetration and damage to tissues [119]. Many nanocarriers have been searched for the use of PDT for the therapy of not only tumors but also infections, skin diseases, and AD (Table **4**) [86, 88-93].

Hirabayashi *et al*. demonstrated the efficacy of a porphyrin molecule combined with a KLVFF peptide fragment in degrading Aβ42 peptides when exposed to UV light, thereby neutralizing the toxicity associated with Aβ42 [120]. A novel derivative of 1,2,4-oxadiazole was recently discovered as an imaging agent for identifying Aβ plaques [121]. Using the affinity of 1,2,4-oxadiazole for Aβ aggregates as a basis, Mangione *et al*. [122] developed a photoreactive oxadiazolic compound with fluorine, aiming to prevent Aβ40 aggregation when exposed to UV light. Methylene blue (MB) has long been investigated for the treatment of AD due to its considerable solubility in aqueous solutions, minimal toxicity, and ability to cross the BBB [123, 124]. Studies have indicated that MB helps to facilitate the Aβ fibrillation process while preventing the formation of soluble and harmful Aβ oligomers [125]. Aβ oligomers and amyloid fibrils affect neuronal cells differently. While amyloid fibrils disrupt cell membrane integrity, Aβ oligomers interact with specific receptors and activate signaling pathways leading to apoptosis [126-129].

Ni *et al*. suggest that the amyloid cascade hypothesis requires revision to focus on toxic oligomers as primary targets [130].

Lee *et al*. [131] reported that under light conditions, MB monomers produce ^1^O_2_ *via* the type II photochemical pathway, which inhibits Aβ aggregation and dissociates pre-existing aggregates. Furthermore, Necula *et al*. suggest that the compound MB exerts its effects by preventing toxic oligomer formation and enhancing fibril creation, highlighting its potential as a therapeutic agent. MB inhibits oligomerization at lower concentrations than Aβ42 monomers but does not completely inhibit Aβ42 aggregation. Instead, it promotes the formation of aggregates that initiate fibril formation. Increasing MB concentrations shortens the fibril nucleation lag time and, at high concentrations, abolishes the nucleation-dependent behavior, thereby promoting fibrillization. MB is thought to stabilize prenuclear assembly-competent intermediates and inhibit oligomerization by depleting the monomer pool. Additionally, MB's ability to promote fibril formation in preformed oligomers indicates that its effect is not limited to monomers. MB also facilitates filament elongation and aids in the formation of fibril bundles.

In conclusion, MB inhibits Aβ42 oligomerization while promoting fibril formation. Since Aβ42 fibrillar species are less toxic and may even be protective compared to oligomers, modulating aggregation through this mechanism has been proposed as a pharmacologically attractive approach [125]. Furthermore, research in animal models has demonstrated the efficacy of MB in ameliorating behavioral deficits and reducing brain amyloid aggregation within the brain [121, 123]. Despite promising findings, a phase III clinical trial conducted by TauRx Pharmaceutical Ltd. revealed that MB failed to decelerate AD progression [132]. Nonetheless, a novel approach has recently emerged, suggesting the utilization of MB as a light-sensitizing agent for phototherapy in AD [121]. In 2017, Lee *et al*. used methylene blue, which disintegrates Aβ42 fibrils under red light irradiation at 630 nm wavelength [111]. Circular dichroism, Thioflavin T (ThT) fluorescent, and native gel electrophoresis assays showed that photoexcited methylene blue demonstrated high effects on inhibition of Aβ-aggregation formation compared to the negligible effect of static methylene blue and control. Utilizing the Drosophila AD model, the anti-Aβ action of light-stimulated methylene blue fully restored the AD phenotype *in vivo*. Photoexcited methylene blue, due to produced singlet oxygen, reduced vacuoles in the brain by ~20%, improved motility, and decreased synaptic toxicity in the neuromuscular junction (NMJ), which has shown *via* confocal microscope and crawling path, and distance analyses compared to the negligible effects of static methylene blue and control. In particular, methylene blue showed a high value of BBB penetration [111].

In 2016, Zhang *et al*. produced a chlorine e6- tanshinone I containing micelles (TAS-Ce6) consisting of a (PEG-*b*-PDPA) diblock copolymer. Tanshinone I (TAS) was loaded on micelles *via* hydrophobic interaction and acted as a molecular Aβ inhibitor, and chlorine e6 (Ce6), a phototransduction agent (like PS), was also able to prevent Aβ fibrillation by releasing TAS under NIR irradiation. The survival rate of PC12 cells significantly increased up to 90% when treated with TAS-Ce6 micelles under a 655 nm laser [133].

In 2017, Chung *et al*. studied carbon dots coated with branched polyethylen-imin (bPEI@CDs) to inhibit the self-assembly of Aβ42 peptides. The branched polyethylene-imin (PEI), which positively influenced the zeta potential value of the nanostructure, interacted with the negatively charged residues of Aβ peptides. ThT analyses and atomic force microscopy (AFM) images demonstrated that under light, bPEI@CDs inhibited the formation of Aβ aggregates and also revealed strong Aβ disaggregation efficacy of more than 50% compared to dark and solo light conditions. These results were also supported by the cell viability assay of PC12 cells [134].

Upconversion nanoparticles doped with lanthanide (UCNPs) have been investigated for their potential in PDT for AD. These UCNPs exhibited high stability, the ability to be excited by NIR light, a large anti-stokes shift, advanced emission, and regulable excitation [135, 136]. The Aβ-targeting peptide KLVFF and fullerene-conjugated UPCN were designed in 2018. Fullerene has a unique dual role in the dark, where it can act as a ROS scavenger and as a potent ROS producer when exposed to visible or UV light. They reported that without light, the UCNP@Fullerene-KLVFF nanostructure showed a protective effect against oxidative stress in Caenorhabditis elegans (CL2006) and increased survival time. Under NIR light, this Aβ-targeted nanoplatform generated ROS and achieved Aβ photooxygenation, which can inhibit Aβ aggregation and reduce the associated neurotoxicity [135].

Porphyrinic metal-organic frameworks (PMOFs), which are stable under physiological conditions and have good biocompatibility, are used as Cu II chelating and photooxidation agents to prevent aggregation. In 2019, Yu *et al*. designed Hafnium-MOFs modified with an Aβ-targeting peptide, LPFFD, for the purpose of both reducing Aβ aggregation and inflammation by chelating Cu ions and inhibiting targeted Aβ aggregation by conjugating Aβ targeted peptides. Transmission electron microscopy (TEM) images and PC12 cell viability assays demonstrate that Hafnium-MOFs inhibit Aβ aggregate. Additionally, Hafnium-MOFs successfully inhibited Aβ aggregates in the CL2006 strain under light. They significantly prolonged their survival compared to no Hafnium-MOFs with light, no Hafnium-MOFs without light, and Hafnium-MOFs without light [137].

In 2022, Ma *et al*. designed PEG-modified copper-cysteine nanoparticles. The PEG modification was carried out to reduce particle size and cytotoxicity, and the copper-cysteine structure was also preferred because of its good physical stability and biocompatibility. Degradation of Aβ aggregation was achieved by 365 nm UV radiation applied to this structure, which has been demonstrated *via* ThT fluorescent assay and AFM images [138].

### 6.2 Photothermal Therapy Studies in Alzheimer's Disease

PTT may be a promising treatment option for AD. Studies, including nanocarriers, have been performed in this area.

In 2018, Ruff *et al*. prepared a nanostructure in which empty gold was functionalized with the Aβ-targeting peptide CLPFFD and PEG-CLPFFD-conjugated gold nanorods. Nanostructures have been irradiated with an 808 nm, 450 mW continuous laser for 2 hours with increasing heat from 30°C to 42°C. Irradiation enhances the inhibitory effect on aggregation, mainly in the case of HauNS, as demonstrated *via* ThT fluorescent assays [145].

Natural metalloproteases, such as insulin-degrading enzymes, matrix metalloproteinase, and neprilysin, provide Aβ degradation. In 2019, Ma *et al*. constructed a NIR-controlled high-throughput artificial metalloprotease, a molybdenum disulfide nanolayer, and a cobalt complex (MoS_2_-Co), inspired by these enzymes. In this study, NIR-MoS_2_-Co nanoplatforms interacted with Aβ monomers and impeded β-sheet configurations (decreased levels of β-sheet content from 71.7% to 27.6%), as shown by circular dichroism. In addition, the heat generated under the 808 nm NIR laser enhanced the MoS_2_-Co hydrolysis process. By NIR irradiation, Aβ monomers were separated into Aβ 1-12, and Aβ 1-20 fragments by MoS_2_-Co, as demonstrated *via* mass spectrophotometry, and this conclusion was also supported by sodium dodecyl sulfate-polyacrylamide gel electrophoresis (SDS-PAGE) results. In addition, mature Aβ fibrils were significantly degraded by the combination of photothermal and hydrolytic effects, as demonstrated *via* Turbidity and dynamic light scattering assays. These results have also been represented by the PC12 cell viability assay [146].

In 2019, Sudhakar and Mani developed silver triangular nanoplates capped with poly(vinyl)pyrrolidone and reported that due to their plasmonic photothermal properties, they were able to cleave preformed Aβ fibrils within 1 hour under 800 nm NIR irradiation at 200 mW. This is a significant improvement compared to the over 70 hours required for an equal concentration of silver spherical nanoparticles under NIR irradiation to achieve similar results. AgTNPs inhibit the formation of Aβ fibrils and dissolve Aβ fibrils. These results have been demonstrated *via* AFM, TEM, and (Attenuated total reflectance-Fourier transform infrared spectroscopy (ATR-FTIR). The AFM images show that pure Aβ protein, when incubated for 24 hours, exhibits mature fibrils, consistent with the results of the Congo red assay. Samples treated with 30 nM silver spherical particles (AgSPs) demonstrate some reduction in fibril formation but still show significant aggregation. In contrast, samples treated with 30 nM silver triangular nanoparticles (AgTNPs) display markedly fewer fibrils, indicating better inhibition of fibrillation. Overall, it can be concluded that AgTNPs are more effective than AgSPs in preventing Aβ fibril formation. The TEM images of samples incubated with different concentrations of AgTNPs show that mature fibrils are entangled throughout the control sample. Additionally, it indicates that increasing the concentration of AgTNPs reduces fibril content more effectively than AgSPs. The Congo red assay and TEM studies indicate that AgSPs and AgTNPs are promising candidates for inhibiting fibril formation and dissolving mature fibrils. The results show that mature fibrils are toxic, with only 30% of cells remaining viable after 24 hours. According to cell viability experiments on SH-SY5Y and BE-(2)-C cells, treating the cells with AgTNP-incubated Aβ fibrils enhanced their viability from 33 to 70% when compared to mature Aβ fibrils [147].

In 2020, Liu *et al*. designed an Aβ targeted nanosystem with enhanced BBB penetration for both PDT and PTT. The system, composed of the Aβ-targeting peptide KLVFF and the FDA-approved porphyrin derivative triphenylporphyrin, demonstrated efficacy in inhibiting Aβ aggregation *in vivo*. The nanoparticles not only improved BBB permeability but also exhibited highly selective photooxygenation of Aβ without affecting other target proteins. When excited by a laser, these nanoparticles transformed from a spherical to an amorphous form upon encountering Aβ, shifting from photothermal to photodynamic activity and thereby inhibiting Aβ aggregates. *In vivo* experiments showed that these nanoparticles reduced Aβ-induced cytotoxicity and extended the lifespan of transgenic *Caenorhabditis elegans* CL2006 [148].

A polydopamine-ruthenium nanosystem (PDA-Ru) was created by Liu *et al*. in 2022. It was formulated as a hydrogen peroxide catalyst, ROS scavenger, and NIR PTT reagent. The photothermal conversion efficiency and catalase activity of PDA were enhanced by integrating Ru nanoparticles. PTT study has been carried out with 808 nm NIR laser (0.5 W/cm^2^) irradiation for 10 min both *in vitro* and in AD model APP/PS1 mice. After receiving laser radiation for 10 min, the temperature of the PDA-Ru solution increased to 43.2°C, which was 5.3°C higher than the PDA solution. The result indicates that PDA-Ru has better photothermal conversion performance than PDA. When NIR light was applied, PDA-Ru was shown to inhibit Aβ fibrils in the TEM images, ThT fluorescence test, and ThS staining test of SH-SY5Y cells. *In vivo*, experiments in AD model APP/PS1 mice showed that NIR-applied PDA-Ru could effectively decrease Aβ deposition and restore microglia’s neuroregulatory function in BV2 cells. Ultimately, neuroinflammation and memory impairments in AD model APP/PS1 mice were improved by NIR-applied PDA-Ru (*p* < 0.05) [149].

## 7 IMAGING AND NANOTHERANOSTIC APPRO-ACH

The term “theranostic” has been used for agents having therapeutic, diagnostic, and imaging properties. Nanotheranostics are nanosized theranostics. Theranostic is a combination of diagnostic and therapeutic applications into a single agent. In this way, diagnosis, imaging, and therapy of diseases can be performed by this single agent, and monitoring of treatment response can also be achieved [150, 151].

Different imaging agents have been used for different imaging modalities. Gadolinium and SPIONs can be used in magnetic resonance imaging (MRI), and iodine and boron in computed tomography (CT), which are anatomical imaging modalities. While Positron-emitting radiopharmaceuticals can be used for positron emission tomography (PET), gamma radiation-emitting radiopharmaceuticals can be used for single photon emission tomography (SPECT). Some atoms, such as fluorescent dyes, including PSs, can be used for NIR fluorescent (NIRF) imaging, and luciferin can be used for bioluminescent imaging as optical imaging. The development of NIRF imaging as optical imaging and NIR dyes has been influenced by fluorescence imaging. Deep tissues can be monitored more effectively with NIR light [152]. For all imaging modalities, the signal is formed by the interaction of activatable probes with their targets [153-158].

Although not all of these methods are routinely used in clinics for the diagnosis and imaging of AD, specific contrast/radiocontrast agents have been searched to obtain an accurate and sensitive diagnosis and imaging of AD. For the diagnosis of AD, the tissue and volume of the brain are examined through structural anatomical imaging with MRI. Cross-sectional images of the brain can be viewed as CT functional imaging. Functional changes, such as cellular or chemical changes, can be followed with targeted radiotracers by SPECT or PET molecular imaging. Optical Imaging is used mainly for experimental purposes today. The diagnosis and drug treatment of AD have usually been carried out separately. More recently, the integration of diagnosis, imaging, and therapy with nanomaterials to develop new therapeutic strategies may also show potential for a new avenue for nanotheranostics. With a simultaneous or combined approach, the pathophysiology of AD, which has not yet been fully understood, may be understood better, and a personalized and efficient approach may be potentially obtained with a single approach (Table **5**).

In 2017, Li *et al*. aimed to increase the inhibition efficiency of Aβ aggregation by designing multifunctional nanomaterials. First, the Aβ15-20 peptide was modified with the Ac-QKLVFF-NH2 sequence, and gold nanorods (AuNR) were combined with polyoxometalates (POMs), which aim to prevent Aβ aggregation based on electrostatic interactions. The change in absorbance of the AuNR was used to monitor Aβ aggregation. The application of AuNR in monitoring the ex-situ kinetic process of amyloid fibrosis is made possible by their distinct absorbance behaviors in the presence of native and fibrillar forms of Aβ1-40. POMs and the Aβ targeting peptide Aβ15-20 also detected Aβ aggregation. When exposed to NIR irradiation, the synthesized AuNR dissolved amyloid deposits in both buffer and mouse CSF successfully suppressed Aβ aggregation and shielded cells from the toxicity of Aβ when exposed to NIR irradiation. This multifunctional nanomaterial demonstrated both nanocarrier, PTT, and nanotheranostic properties [159].

For the detection and photothermal degradation of produced Aβ fibrils, Liu *et al*. developed dual-conjugated AuNR containing APH ST0779 and Aβ oligomers and fibril recognition-binding fragment (scFv 12B4) (GAS) in 2019. In this study, AuNR loaded with single-chain variable fragment (scFv) 12B4 and thermophilic acylpeptide hydrolase (APH) ST0779 were developed as a smart theranostic complex possessing NIR absorption properties. This complex enabled rapid detection of Aβ aggregates as well as effective degradation of Aβ aggregates and inhibition of Aβ-mediated toxicity through NIR PTT. However, it is challenging for *in vivo* applications due to its low efficiency in terms of enzyme activity, low stability, and rapid proteolytic degradation. ScFv 12B4 has a low molecular weight and can identify and bind to Aβ oligomers and fibrils across the BBB, but is ineffective as an Aβ modulator due to its short half-life. The stability of APH ST0779 was improved by immobilizing APH ST0779 and scFv 12B4 on AuNR. More importantly, the photothermal activity of AuNR under NIR light enabled the disaggregation of Aβ fibrils and the activation of the thermozyme, which accelerated the degradation of Aβ. The theranostic GAS, which detected Aβ aggregation in real time, displayed an increased effect in dissociating Aβ aggregates, reducing neurotoxicity caused by Aβ induced and delayed Aβ aggregation paralysis in the strain CL4176, which is a transgenic nematode [160].

In the laboratory, GAS has been shown to inhibit and disperse Aβ accumulation and to reduce haem-Aβ peroxidase-like activity. When applied to cultured cells, GAS administration reduced Aβ-induced cytotoxicity and delayed Aβ-induced paralysis in the CL4176 C elegans model of AD. Additionally, the photothermal influence of GAS under NIR laser exposure assisted in dispersing Aβ accumulations and amplifying APH activity to eliminate Aβ. Functioning as a targeting sensor and inhibitor, GAS enabled real-time identification of Aβ accumulations [160].

Ultrasmall superparamagnetic iron oxide nanoparticles (USPIONs) are a promising class of nanoparticles employed in biomedical research because of their great biocompatibility and versatility as MRI contrast agents. In 2020, Cai *et al*. prepared ultrasmall superparamagnetic iron oxide nanoparticles that included an NIR fluorescent dye-N-hydroxysuccinimide as a multimodality contrast agent. Cai *et al*. performed NIR fluorescence and MRI of Aβ structures in the brain of APPswe/PSEN1dE9 transgenic mice simultaneously, and in this way, they differentiated Aβ fibrils [52].

In 2021, Gong *et al*. developed a theranostic nanocaptor, designated as BfeCN, by conjugating carbon nitride (C_3_N_4_) nanodots to Fe_3_O_4_@mesoporous silica nanospheres and incorporating benzothiazole aniline (BTA) on the surface. This nanocaptor was designed for targeted therapy and PTT while exhibiting theranostic properties. Nanocaptors are nanosystems that typically bind metal ions to form chelates, allowing them to capture metals both *in vitro* and *in vivo*. The Fe_3_O_4_ core serves as a high-resolution MRI probe, enabling the diagnosis and monitoring of AD. The nanoparticles demonstrated photothermal activity, enhancing their ability to cross the BBB under NIR laser illumination, as shown by both *in vivo* and *in vitro* studies. Due to the high affinity of BTA for the β2 position of Aβ fibrils, the BTA-modified nanocaptor specifically targeted Aβ plaques and provided fluorescence imaging for sensitive detection of Aβ aggregates. The C_3_N_4_ nanodots protected against copper ion toxicity inhibited Aβ aggregate formation, and acted as a chelator for free Cu ions. The BfeCN nanocaptor targeted Aβ plaques through magnetic targeting by the Fe_3_O_4_ core while also generating heat to dissolve pre-existing Aβ aggregates. Additionally, C_3_N_4_ nanodots captured excess copper ions, preventing the formation of Cu^2+^-Aβ complexes and thereby clearing Aβ aggregation [161].

In 2022, Sharma *et al*. produced self-fluorescent single tryptophan nanoparticles (TNPs) as a nanotheranostic approach for AD. Due to their autofluorescent properties, TNPs were shown to strongly inhibit and disrupt both fibrils formed from the amyloid dipeptide phenylalanine-phenylalanine (FF) and the Aβ 42 peptide in cellular studies using SH-SY5Y cells, as observed *via* confocal microscopy. These nanostructures protected neurons from neurotoxicity caused by Aβ 42 peptide and FF aggregates while proving nontoxic to the neurons themselves. In animal studies, TNPs improved learning and spatial memory in rats with an intracerebroventricular streptozotocin-induced AD phenotype [162].

Nanotheranostics might contribute to diagnosing and addressing the pathophysiology of AD, which is not yet fully understood, and provide personalized and effective treatment with a single approach. In this respect, these studies seem very promising. However, most of these studies, which have not yet been tested in higher organisms, are still at the experimental stage and have limited information in terms of toxicological profiles and scale-up requirements.

## CONCLUSION

The limitations of AD therapy include the inability to precisely define the disease’s pathophysiology and its rapid progression. The disease often reaches an advanced stage by the time symptoms appear. Therefore, early diagnosis and imaging of AD by using sensitive imaging modalities such as nuclear imaging, radiological imaging, and optical imaging with target-specific contrast agents may contribute to identifying physiological changes before symptoms manifest and enable early intervention. To address these challenges, target-specific nanomaterials capable of penetrating the BBB are being developed for AD diagnosis and/or therapy. PDT and PTT offer potential non-invasive approaches for AD treatment. Recently, the integration of PDT with nanoplatforms for diagnosis, imaging, and novel therapeutic strategies has shown potential for advancing nanotheranostics. This approach may provide a better understanding of AD pathophysiology and enable personalized and effective therapies. Despite these advances, such approaches remain at the research level and are in the early stages of becoming effective treatments. PS agents require further analysis to reduce their cytotoxic potential to healthy tissues before they can be considered viable for AD treatment.

## Figures and Tables

**Table 1:** Approved drugs for Alzheimer's disease [45-47].

**Name (Generic/Brand) Indicated for**	**Name (Generic/Brand) Indicated for**	**Treatment**	**Authorities**
Aducanumab Aduhelm^®^	Alzheimer's disease (MCI or mild dementia)	Changes disease progression	FDA, EMA-(Application withdrawn)
Donanemab Kisunla™			FDA
Lecanemab Leqembi^®^			FDA, EMA-opinion under re-examination
Donepezil Aricept^®^	Mild to severe dementia due to Alzheimer’s	Treats cognitive symptoms (memory and thinking)	FDA, EMA, Canada
Galantamine Razadyne^®^	Mild to moderate dementia due to Alzheimer’s		FDA, EMA, Canada
Memantine + Donepezil Namzaric^®^	Moderate to severe dementia due to Alzheimer’s		FDA, EMA
Memantine Namenda^®^			FDA, EMA, Canada
Rivastigmine Exelon^®^	Mild to moderate dementia due to Alzheimer’s or Parkinson’s		FDA, EMA, Canada
Brexpiprazole Rexulti^®^	Agitation associated with dementia due to Alzheimer's disease	Treats noncognitive symptoms (behavioral and psychological)	FDA, EMA
Suvorexant Belsomra^®^	Insomnia, has been shown to be effective in people living with mild to moderate Alzheimer’s disease		FDA

**Table 2:** Nanocarrier-based systems for Alzheimer's disease [72].

**Nanocarrier-based Systems**	**Carrier Material**	**Potential Drug Candidate**	**Investigation Model**	**Target**	**References**
Liposomes	PLGA - modified with anti-transferrin receptor monoclonal antibody and Aβ-inhibitor	Peptide Aβ	Porcine brain capillary endothelial cells	Aβ-inhibition	[73]
Gold (Au) NPs	Gold colloids - rods and spheres	CLPFFD peptide	Porcine brain capillary endothelial cells	Aβ-inhibition	[74]
	Gold nanoparticles	Tetrapeptide from maize	KM mice. Morris water maze, rotarod performance, and autonomic activity behavioral tests and PC12 cell line viability	Oxidative stress/ Cholinergic systems	[75]
Micelles	(PEG)-*b*-PCL) co-assembled (PAE-*b*-PCL) micelles	VQIINK peptide	Okadaic acid-induced AD model mice. Behavioral experiments, including novel object recognition tasks. Morris water maze tests and N2a cell line viability	Inhibition of tau aggregation	[76]
Mesoporous silica NPs	N-Cetyl trimethyl ammonium bromide - modified with succinic anhydride and 3-aminopropyltriethoxysilane	Rivastigmine hydrogen tartrate	Mimicked gastrointestinal and body fluids and neuroblastoma SH-SY5Y cell line viability	Neuroprotection/ Cholinergic systems	[77]
Self-nano-emulsifying drug delivery systems	Black seed oil, Transcutol P, Cremophor RH40, Labrasol SNEDDs	Curcumin-Piperine	AD model. Albino mice behavioral experiments, including novel object recognition tasks and Morris water maze tests	Learning and memory enhancement	[78]
Graphene quantum dots	*Clitoria ternatea* mediated synthesis of graphene quantum dots	*Clitoria ternatea* Graphene quantum dots (ctGQDs)	In rats, ctGQDs demonstrated enhanced learning and memory in the water Morris maze tests	Cholinergic systems	[79]
Carbon nanotube	Single-walled carbon nanotube	Tunable zero-dimension	Coarse-grained nanoparticle model and implicit/explicit lipid models	Inhibition of Aβ and cholinergic systems	[80]

**Abbreviations**: PEG: Polyethylene Glycol, PLGA; Poly(lactic-co-glycolic acid), CLPFFD; Cysteine-Leucine-Proline-Phenylalanine-Phenylalanine-Aspartic Acid.

**Table 3:** Commonly used photosensitizers.

**Photosensitizer**	**Activating Wavelength**	**Advantages/Disadvantages**	**References**
Rose Bengal	525 nm	Photoexcited rose bengal protected Aβ from transition to β-sheet structure by blocking an early step in the self-assembly pathway, has minimal cytotoxicity, biocompatible/ poor lipophilicity, short half-life (for AD).	[105-107]
Indocyanine green	800 to 830 nm	Indocyanine green is approved by the US Food and Drug Administration as a NIR clinical imaging agent/ the limited duration of effectiveness (for carcinoma).	[108, 109]
Toluidine blue O	630 nm	Toluidine blue O molecules have a high ability to bind to DNA and could aggregate alone on the DNA surface/clearing of the blue color of Toluidine blue O, from the culture media (for antiprotozoal).	[110]
Methylene blue	600 to 660 nm	Methylene blue has a high quantum yield of ^1^O_2_ generation (ϕΔ~0.5) under red light, light-induced inhibition effect on Aβ42 aggregation, high solubility, minimal toxicity, high BBB penetration/Since its stability in sunlight is limited, light-proof containers should be used when preparing samples (for AD).	[111]
Acridine orange	490 nm	Easy to understand apoptosis, active and inactive reproductive cells, and pH gradients within the cell thanks to its special staining/ Sensitive to pH levels (for carcinoma).	[112]
Photofrin	630 nm	The most commonly used photosensitizer/long-term photosensitive reactions on patients' skin (for carcinoma).	[113]
Chlorin e6	650 to 700 nm	It produces high ROS, is FDA-approved, and is effective against a broad spectrum of cancer/ Low biodistribution and rapid clearance due to its high hydrophobicity (for carcinoma).	[114]
Psoralen	320 to 400 nm	It has intrinsic anti-inflammatory properties, which can enhance the therapeutic outcome for patients with chronic skin conditions/it has an increased risk of skin damage like unpredictable bullous phototoxic reactions (for dermatology).	[115, 116]
Azulene	625 nm	High intrinsic anti-inflammatory activity/photomutagenicity possibility (for dermatology).	[117, 118]

**Table 4:** PDT studies in Alzheimer's disease.

**PS**	**Results**	**Biological Model**	**References**
TAS-loaded Ce6 micelles	By ThT fluorescence measurements, TAS-loaded Ce6 micelles show inhibiting Aβ monomers from formation Aβ aggregates and degradation of protofibrills to small fragments. Also, *via* laser irradiation, TAS-loaded Ce6 micelles show an additional reduction of 90%, confirming the complete degradation of Aβ fibrils.	PC12 cell/Aβ fibrils	[133]
Methylene blue	MB shows inhibition of aggregate formation and inhibition of preformed aggregates and high affinity for AB 42 peptides while showing a 50% reduction in AB aggregation under red light (by PDT effect) compared to dark conditions. MB also reveals reduced synaptic toxicity and neuron damage in the drosophila AD model.	Drosophila/Aβ fibrils	[111]
bPEI-Carbon Dots	bPEI@CDs suppress the Aβ aggregation process *via* electrostatic interactions. Photoactivated bPEI@CDs show a more pronounced effect on Aβ aggregation and dissociation of β-sheet-rich assemblies *via* the generation of reactive oxygen species.	PC 12 cells/Aβ fibrils	[137]
UCNP@Fulleren-KLVFF	UCNP@C60-pep has dual effects with its ROS producer properties under NIR and ROS scavenger properties in the dark. It shows low phototoxicity in non-target areas by binding directly to Aβ peptides with targeting peptides. After NIR light excitation, PC12-Aβ cells incubated with UCNP@C60-pep for 24 hours gave similar ROS values ​​to pc12 cells, suggesting effective ROS quenching. UCNP@C60-pep extends the lifespan of CL2006 and mitigates paralysis caused by Aβ toxicity.	*Caenorhabditis elegans* (CL2006)/ Aβ fibrils	[135]
Hf-MOF	Porphyrinic metal-organic frameworks, particularly Hf-MOFs, serve as effective Aβ photo-oxidants in the treatment of AD. Hf-MOFs inhibit Aβ aggregation and, when modified with the Aβ-targeting peptide LPFFD, enhance cellular targeting of Aβ, thereby reducing Aβ-induced neurotoxicity. *In vivo*, studies indicate that these well-designed LPFFD-modified Hf-MOFs can decrease Aβ-induced neurotoxicity and extend the lifespan of the commonly used transgenic AD model, Caenorhabditis elegans CL2006.	*Caenorhabditis elegans* (CL2006)/ Aβ fibrils	[137]
BAP-1 based photocatalyst	The photocatalyst offers a non-invasive method, passage through the BBB, and selective binding to Aβ aggregates. By enhancing microglia degradation, the photocatalyst facilitates the clearance of Aβ aggregates from the brains of living AD model mice. Increases cell viability of PC12-Aβ cells.	PC12 cells, App knockin mouse	[139]
PEG-Co-Cy nanoparticles	A novel theranostic photosensitizer, copper cysteine (Cu-Cy), modified with polyethylene glycol (PEG), for AD treatment. The PEG modification reduced particle size and cytotoxicity. Under UV irradiation, Cu-Cy-PEG effectively produced reactive oxygen species (ROS) and degraded Aβ(1-40) aggregates *in vitro*, converting them into amorphous particles. These results indicate that Cu-Cy-PEG could be a promising therapeutic option for AD.	Aβ fibrils	[138, 140]
[Ru(bpy)3]^2+^	A ruthenium (II) complex, [Ru(bpy)3]^2+^, is a photoresponsive anti-Aβ agent that drives the dissociation of β-amyloid (Aβ) aggregates into small, nontoxic fragments under visible light. Through various analyses, it was demonstrated that [Ru(bpy)3]^2+^ effectively destabilizes the β-sheet structure of Aβ aggregates when illuminated with white light. The mechanism involves oxidative damage to Aβ peptides caused by reactive oxygen species generated from the photoexcited complex. Additionally, [Ru(bpy)3]^2+^ inhibited Aβ monomer self-assembly at concentrations as low as 1 nM and reduced the cytotoxicity of Aβ aggregates. These findings highlight the potential of [Ru(bpy)3]^2+^ in Alzheimer's disease therapy.	PC12 cells	[140]
Catalyst 9	Catalyst 9 provides a non-invasive method as it can oxygenate Ab embedded under mouse skin. When activated by NIR light, Catalyst 9 effectively penetrates biological tissues and BBB, shows high selectivity for aggregated Aβ, successfully reduces intact Aβ levels in the brains of AD model mice, and increases cell viability of PC12-Aβ cells.	PC12 cells, App knockin mouse	[141]
Zn phthalocyanine	A thymine-modified Zn phthalocyanine (T-ZnPc) designed to specifically recognize and bind to elevated levels of Fe^3+^ and Al^3+^ around amyloid beta (Aβ) protofibrils in AD. The results showed that T-ZnPc enhances its photodynamic therapy (PDT) activity, generating ROS that effectively degrade Aβ protofibrils (62% degradation with Al-T-ZnPc and 81% with Fe-T-ZnPc). Additionally, T-ZnPc inhibited the formation of new Aβ protofibrils and reduced free Fe^3+^ and Al^3+^ concentrations in the brain, which may further aid in Alzheimer’s treatment.	PC12 cells	[142]
Catalyst 7	Provides a non-invasive method to prevent disruption of brain tissues and effectively permeate the BBB; selectively degrade aggregated Aβ proteins and significantly decrease the concentration in AD model mice thanks to chronic administration, increasing cell viability of PC12-Aβ cell.	PC12 cells, App knockin mouse	[143]
Ce6	Provides an effective photodynamic treatment for selectively targeting amyloid beta (Aβ) oligomers in AD. Catalytic amounts of photoexcited chlorin e6 inhibit Aβ aggregation and toxicity and can reverse the aggregation process in the dark by binding to soluble oligomers. It selectively damages Aβ histidine residues (H6, H13, and H14) and induces cross-linking through singlet oxygen generation without affecting other proteins like ubiquitin and α-synuclein. Additionally, chlorin e6 inhibits Cu^2+^-induced Aβ aggregation and acts as a chelator in darkness.	PC12 cells	[144]

**Note**: Ru(bpy)3] 2^+^: tris(2,2-bipyridine) ruthenium (II), Cranad-2: Catalyst containing one difluoro boron 1,3-diketonate (electron acceptor) and two N, N-dimethylaniline (electron donor) moieties, Ce6: ChlorinE6, BAP-1: Boron-dipyrromethene based photocatalyst.

**Table 5:** Nanotheranostic research in Alzheimer's disease.

**Delivery System/ Nano Theranostic**	**Biological Model**	**Comment**		**References**
		**Imaging**	**Therapy**	
Gold Nanorod/ Aβ15-20-POM-AuNR	S4880202 Normal Mice/PC 12 cell	- Aβ15-20 is an Aβ-targeting element. - The different absorbance behaviors of AuNR with the single and fibrillar forms of Aβ1-40 enable their use to monitor the ex-situ kinetic process of amyloid fibrosis.	- Aβ15-20 and POM are inhibitors of Aβ. - AuNR with NIR irradiation generates local heat. - NIR-irradiated nanosystems prevent Aβ aggregation and dissociate amyloid deposits in mice CSF, shielding cells from the toxicity of Aβ.	[159]
Gold Nanorod/ AuNR loaded with scFv 12B4 and thermophilic APH ST0779	*C. elegans/* SH-SY5Y cells	- Anti-Aβ scFv 12B4 is an Aβ-targeting element. - The different absorbance behaviors of AuNR with the single and fibrillar forms of Aβ1-40 enable their use to monitor the ex-situ kinetic process of amyloid fibrosis.	- APH disrupts Aβ monomers with the Aβ inhibitory and targeting effects of scFv, dissociates Aβ fibrils, and attenuates Aβ-mediated peroxidase activity. - AuNR with NIR irradiation generates local heat. - Nanosystem with NIR irradiation inhibits Aβ aggregation and dissociates amyloid deposits. This reduction in neurotoxicity is observed in *C. elegans* both *in vitro* and in cultured cells.	[160]
Super paramagnetic-ferrumoxyde-nanoparticles/ DPA-PEGylated USPIONs-ph1/ph2	SH-SY5Y cell	- DPA acts as a fluorescent probe. - SPIONs are magnetic probes.	- DPA is an inhibitor of aggregation. - SPIONs act as an inhibitor of Aβ aggregation. - There is a significant difference between double transgenic and wild-type control mice on NIR fluorescence and *in vivo* magnetic resonance imaging, and specific binding to Aβ plaques was further confirmed by histological staining in brain slices. They prevented Aβ seeding-mediated aggregation and formation of toxic Aβ species. - They alleviated cytotoxicity by reducing Aβ1-42 in SH-SY5Y cells.	[52]
Mesoporous silica nanosphere-nanodot/ Carbon nitrite (C_3_N_4_) nanodots to Fe_3_O_4_ @mesoporous silica nanospheres-BTA	Double transgenic APP/PS1 mice/ BV-2 cells	- The Fe_3_O_4_ core functions as an MRI probe that allows the diagnosis and screening of AD. - Along with the photoluminescence feature of CTA, nanocaptor increases its effectiveness in the treatment of AD by using MRI and FL imaging together. - BTA is an Aβ-targeting element, and the Fe_3_O_4_ core has magnetic targeting properties.	- As chelators, the C_3_N_4_ nanodots effectively limit the formation of Aβ aggregates and provide neuroprotection against the toxicity associated with copper ions. - By capturing copper ions and photothermal properties (the Fe_3_O_4_ core generates local hyperthermy under NIR irradiation and also owing to a magnetic field), it reduces. Aβ-induced neurotoxicity in mice and cultured cells and corrects impaired memory in double transgenic APP/PS1 mice.	[161]
Organic nanoparticle/ Tryptophan nanoparticles	Sprague-Dawley (SD) rats/ SH-SY5Y	- Tryptophan is an autofluorescent molecule that enables bioimaging due to its nature.	- Tryptophan acts as an inhibitor of FF and Aβ aggregation. - It reduces cell death in SH-SY5Y and cognitive deficits and suppresses Aβ42 oligomer accumulation in the brains of ICV-STZ-induced AD mice due to its role in 5-HT synthesis.	[162]

## LIST OF ABBREVIATIONS

αAPP: APP Fragment Cleaved by α-secretase
AGEs: Advanced Glycation End Products
APOJ: Clusterin
APP: β-amyloid Precursor Protein
BACE1: β-site APP Cleaving Enzyme 1
BTA: Benzothiazole Aniline
CP: Choroid Plexus
CSF: Cerebrospinal Fluid
GAS: AuNR Included APH ST0779 and Aβ Oligomers and Fibrils Recognizer-Binder Fragment (scFv 12B4)
GQDs: Graphene Quantum Dots
lgG1: Immunoglobulin Gamma 1
LRP-1: Low-density Lipoprotein Receptor-related Protein 1
mAChRs: Muscarinic Acetylcholine Receptors
MOFs: Metal-organic Frameworks
nAChRs: Nicotinic Acetylcholine Receptors
PCL: Poly-capro Lactone
PEI: Poly-ethylen-imin
PICALM: Phosphatidylinositol Binding Clathrin Assembly Protein
POMs: Polyoxometalates
RAGE: Receptor for Advanced Glycation End Products
ROS: Reactive Oxygen Species
SNEDDs: Self-nanoemulsifying Drug Delivery Systems
SOD-1: Superoxide Dismutase Type 1
SPECT: Single Photon Emission Tomography
SPION: Superparamagnetic Iron Oxide Nanoparticles
UCNP: Upconversion Nanoparticles
USPION: Ultrasmall Superparamagnetic Iron Oxide Nanoparticles
β-sAPP: APP Fragment Cleaved by β-secretase
PC12-Aβ: Aβ Aggregates Incubated PC12 Cells
